# Use of moxibustion to treat primary dysmenorrhea at two interventional times: study protocol for a randomized controlled trial

**DOI:** 10.1186/s13063-015-0552-1

**Published:** 2015-01-30

**Authors:** Jie Yang, Siyi Yu, Lixing Lao, Mingxiao Yang, JianPing Chen, Xiao Luo, Yongxia Wang, Xiangzhu Chen, Juan Li, Lihua Zhu, Qianhua Zheng, Youping Hu, Xi Wu, Fanrong Liang

**Affiliations:** Chengdu University of Traditional Chinese Medicine, NO.37, Shierqiao Road, Jinniu District, Chengdu, Sichuan 610072 China; School of Chinese Medicine, The University of Hong Kong, 10 Sassoon Road, Pokfulam, Hong Kong, China

**Keywords:** Moxibustion, Primary dysmenorrhea, Intervention time, Efficacy, RCT, Study protocol

## Abstract

**Background:**

Dysmenorrhea is a common menstrual complaint among adolescent girls and women of reproductive age. The treatment of dysmenorrhea is typically selected from multidisciplinary options, including complementary and alternative medicine such as acupuncture and moxibustion. However, there are few published randomized controlled trials concerning moxibustion treatment for dysmenorrhea. This trial aims to investigate the efficacy and safety of moxibustion for primary dysmenorrhea, and to identify the optimal time of moxibustion treatment for primary dysmenorrhea.

**Methods/Design:**

This protocol is for a randomized controlled trial in which the assessor and statistician will be blinded. A total of 222 eligible patients with dysmenorrhea will be randomly assigned to three groups in a 1:1:1 ratio as treatment group A (treated before menstruation onset), treatment group B (treated at the onset of menstruation), or control group C (waiting list group). The participants assigned to the treatment groups will receive suspended moxibustion treatment at *Sanyinjiao* (SP6) and *Guanyuan* (CV4), while the waiting list group will not receive moxibustion treatment until the completion of the study. The trial period will consist of three baseline menstrual cycles, three menstrual cycles of treatment, and three menstrual cycles in the follow-up period. The primary outcome will be measured by changes in the Cox Menstrual Symptom Scale and the secondary outcomes will be measured using the Visual Analogue Scale, Cox Retrospective Symptom Scale, diary entries, the Self-rating Depression Scale, and the Self-rating Anxiety Scale. The safety of moxibustion will be assessed at every visit.

**Discussion:**

This trial aims to assess the effectiveness and safety of moxibustion for primary dysmenorrhea, as well as to determine whether the optimal time of treatment for primary dysmenorrhea in clinical practice is before or after the onset of menstrual pain.

**Trial registration:**

Chinese Clinical Trial Register: ChiCTR-TRC-14004627, registered on 9 May 2014.

## Background

Dysmenorrhea is a common menstrual complaint among adolescent girls and women of reproductive age [1]. It usually consists of two subcategories: primary and secondary dysmenorrhea. The focus of this paper is primary dysmenorrhea (PD), which is defined as painful menses in women with normal pelvic anatomy. Studies using different measurements have reported the prevalence of PD as between 20 and 90% of adolescent girls [2]. An Australian study found the prevalence of dysmenorrhea varied between 16 and 91%, with symptoms becoming severely painful in 2 to 29% of the women studied [3].

Dysmenorrhea has a significant impact on women’s lives and represents a substantial public health burden [4-6]. Several studies have shown that female individuals with dysmenorrhea report that it affects their academic performance and social and sporting activities, which is a distressing finding given the availability of effective medications [7,8]. Nonsteroidal anti-inflammatory drugs are widely used as first-line therapy for women with dysmenorrhea [9,10]. However, in some women the side effects may not be well tolerated or the pain relief provided may be inadequate. Therefore, a large number of patients with PD are turning to complementary and alternative medicine such as acupuncture and moxibustion [11].

Moxibustion is widely used in Asian countries as a part of Traditional Chinese Medicine (TCM). It is a thermal treatment that involves placing ignited material (usually *Artemisia vulgaris*) near acupuncture points to cause a warm and painless sensation [12]. Moxibustion is used to regulate the meridians and visceral organs of the human body [13]. Suspended moxibustion is commonly used, which refers to the application of a burning moxa stick over the acupuncture points from a distance. In TCM, dysmenorrhea is etiologically caused by the stagnated blood or *Qi* circulation in the uterus. The pathological factors are mostly dampness, blood stasis, and *Qi* obstructions. Moxibustion as a therapy features on the effect of heat and *Artemisia argyi*, and thus can improve blood circulation in the uterus and its surrounding veins, and also help to absorb local pathological discharges in patients with PD. It is considered effective in resolving blood stagnation, *Qi* stagnation, and discomposing cold-damp coagulation, leading to an improved health state [14,15]. Clinical trials and systematic reviews have evaluated the effect of moxibustion for conditions including dysmenorrhea [16], breech presentation [17], ulcerative colitis [18], stroke [19], menopausal hot flashes [20], and constipation [21].

Since ancient times, TCM has prescribed acupuncture and moxibustion to treat dysmenorrhea. Acupuncture stimulation, which involves thrusting or twisting needles, results in various biochemical reactions that can have effects throughout the body; moxibustion uses heat stimulation at various temperatures, from mild skin warmth to tissue damage caused by burning. This heat stimulation can yield inflammatory responses and induce vasodilation by releasing mediators such as histamine and substance P around the site of moxibustion therapy [22,23]. According to TCM, the treatment of PD in clinical practice is usually started before menstruation. Several studies have used different time sequences of acupressure in dysmenorrhea to seek evidence-based medical proof regarding the appropriate point selection and timing of intervention in PD [24]. Unfortunately, clinical trials regarding the optimal time of moxibustion treatment intervention are lacking.

This is a protocol for a randomized controlled trial (RCT) that aims to assess the efficacy and safety of moxibustion, and to determine whether the optimal time of treatment for PD is preventively or after menstrual pain begins.

## Methods/Design

### Setting

This RCT will be conducted in a single center, with the assessor and statistician blinded to treatment allocation. A total of 222 patients diagnosed with PD in accordance with the Clinical Guidelines of Primary Dysmenorrhea by the Society of Obstetricians and Gynecologists of Canada will be enrolled in this trial [25]. The whole treatment process will be carried out in the Third Teaching Hospital of Chengdu University of Traditional Chinese Medicine, Chengdu, Sichuan, China.

### Patients

#### Recruitment strategies

There will be three strategies for PD patient recruitment. First, participants will be recruited from the outpatient and inpatient department of the Third Teaching Hospital of Chengdu University of TCM. Second, printed recruitment posters will be distributed in public clinics and nearby communities. Third, advertisements will be published in the campuses of Chengdu University of TCM, Sichuan University, Southwest University of Nationalities and Southwest Universities of Finance and Economics, and Sichuan Vocational and Technical College of Communications.

#### Inclusion criteria

Participants meeting the following criteria will be included:

1. Aged from 18 to 30 years without history of delivery,
2. Normal menstrual cycle (28 ± seven days) during the last three months,
3. Diagnosed with PD according to the Primary Dysmenorrhea Consensus Guidelines [25],
4. Diagnosed with the TCM pattern of stagnation of *Qi* and blood, or retention of cold-damp coagulation (see Table 1) [26],

**Table 1:** **Traditional Chinese medicine pattern differentiation protocol for women with primary dysmenorrhea**

**Diagnostic patterns**	**Items**
**Diagnostic pattern 1**	
Stagnation of *Qi* and blood	Pain: distended, stabbing abdominal pain before or during the period aggravated by pressure.
	Menstruation: dark purplish in color, clotty and hesitant or scanty in flow, pain relieved after clots discharged.
	Accompanying symptoms: distending in breasts, mood swings.
	Tongue: purplish.
	Pulse: wiry.
**Diagnostic pattern 2**	Pain: distended, stabbing abdominal pain before or during the period, favorably to warmth, lower back pain.
Retention of cold-damp coagulation	
	Menstruation: dark purplish in color, clotty and hesitant in flow, pain relieved after clots discharged.
	Accompanying symptoms: distending in breasts, mood swings, and aversion to cold.
	Tongue: pale and white greasy tongue coating.
	Pulse: deep and slow.
5. Menstrual pain score of over 40 mm on the Visual Analogue Scale (VAS),
6. Agree to keep a dysmenorrhea diary during the study period, and
7. Agree with all procedures in this trial by signing a written informed consent form.

#### Exclusion criteria

Participants meeting one or more of the following criteria will be excluded:

1. Secondary dysmenorrhea caused by endometriosis, uterine myoma, endometrial polyps, pelvic inflammatory disease, or other gynecological problems confirmed by a gynecological abdominal ultrasound B examination;
2. Serious contraindications (for example, a life-threatening condition or progressive central nervous disorder);
3. Any other condition that the investigator judges as likely to make the patient unable to complete, comply, or unsuitable for the study;
4. Uncontrolled diagnosed psychiatric disorders;
5. Intent to have a baby during any period of the trial;
6. Having taken antidepressants, anti-serotonin, barbiturates, or psychotropic drugs in the previous two weeks; or
7. Currently receiving or having received any other treatment for PD in the previous three months.

#### Sample size

A previous study found that the mean on the Cox Menstrual Symptom Scale (CMSS) of PD patients after moxibustion was 1.62 [24]. The average CMSS values of subjects in the proposed study, with the square of standard deviation, are expected to be 1.7, 1.9, and 2.5 for patients treated with moxibustion before menstruation, those treated at the time of menstrual onset, and the waiting list group, respectively. In this case, the three groups are set with the ratio of 1:1:1 as the statistical power is 0.90 and the significance level is 0.05. The sample size was estimated by One Way ANOVA Power Analysis (11th edition, NCSS, LLC, Utah, United States) in NCSS-PASS (11th edition, NCSS, LLC, Utah, United States). After the estimation, the effect size is 0.2266 with 64 patients in each group and 192 patients in total. Therefore, there should ideally be 222 patients in total, with each group containing no less than 74 patients, estimating a dropout rate is 15%.

### Ethics

This study protocol was reviewed and approved by the Institutional Review Board of the Teaching Hospital of Chengdu University of Traditional Chinese Medicine in February 2014 (approval number: 2013KL-034). The present study was financially supported by the National Natural Science Foundation of China and the trial was registered in Chinese Clinical Trials Registry with the identifier number ChiCTR-TRC-14004627. Prior to undertaking any study-related procedures, all participants will provide written informed consent.

### Procedure

An independent assessor will initially assess each potential participant according to the inclusion and exclusion criteria. After completing a screening test, participants will enter a baseline period of three months (three menstrual cycles) without moxibustion treatment, during which they will be asked to record menstrual pain and use of acute medication in a dysmenorrhea diary.

### Randomization

After participants have completed a baseline evaluation, eligible subjects will be randomly classified into three groups: treatment group A (treated before menstruation), treatment group B (treated as the onset of menstruation), and control group C (waiting list group). A computer-generated, blocked random allocation sequence will be generated with SAS software (Version 9.1, SAS Institute Inc., NC, United States). Another research coordinator who is not involved with data collection will provide the random number and group assignment immediately to the independent assessor in opaque, sealed envelopes. This procedure guarantees that randomization concealment is adequate, and not influenced by the acupuncturists or participants. Random allocation will be performed after the first visit to participants who provide informed consent and meet the inclusion criteria. If a participant is eligible to be included in the study, the researcher will open the corresponding envelope in front of the participant. The envelopes will be opened only after the enrolled participants have completed all baseline assessments.

**Figure 1:**
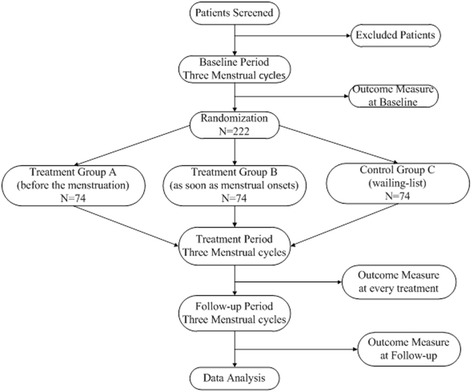
**Flow chart of the trial.** Participants with a diagnosis of primary dysmenorrhea will be recruited at the Third Teaching Hospital of Chengdu University of Traditional Chinese Medicine. All participants should endure a baseline period of three menstrual cycles and inappropriate participants will be excluded. A total of 222 participants will be randomized to three groups: treatment group A (treated before the menstruation), treatment group B (treated at the onset of menstruation), or control group C (waiting list group) in a 1:1:1 ratio. The trial period will consist of three baseline menstrual cycles, three menstrual cycles of treatment, and three menstrual cycles in the follow-up period, and the outcome will be measured at every visit.

Figure 1 summarizes the design of the study and Table 2 outlines the measures used in the trial. The duration of the study for every participant will be nine menstrual cycles, including three menstrual cycles in the baseline, treatment, and follow-up periods, respectively.

**Table 2:** **Study design schedule**

**Period**	**Baseline**	**Inclusion**	**Treatment**			**Follow-up**		
Visit		1	2	3	4	5	6	7
Menstrual cycle	−3 month	0 month	1 month	2 month	3 month	4 month	5 month	6 month
Patients		√						
Inclusion and exclusion criteria		√						
Informed consent		√						
Physical examination		√			√			
Medical history		√						
Comorbidities		√	√	√	√	√	√	√
Outcomes								
CMSS			√	√	√	√	√	√
VAS	√	√	√	√	√	√	√	√
RSS		√						
SDS and SAS		√			√			
Dysmenorrhea diary	√	√	√	√	√	√	√	√
PGF2a, PGE2		√			√			
Gynecological abdominal ultrasound B	√							
Trial evaluation								
Patient’s compliance					√			√
Reasons for dropout or withdrawals					√			√
Adverse events					√			
Safety evaluation								√

### Blinding

Double blinding of the clinician and patient is unfeasible owing to the unique nature of moxibustion operations. However, to ensure the integrity of the trial, the researchers will not assess the efficacy of treatment to avoid misleading the patients and inducing potential impacts on the compliance and outcome measurements. The therapist, the assessors of therapeutic effect, the data manager, and the statistician will be separated. Moreover, according to the principle of blinding, only patients allocated to the waiting list group will be told to wait for free treatments at the end of the study; allocation information will be strictly withheld from patients in the other two groups.

### Interventions

Acupuncturists must receive a license from the Ministry of Health of the People’s Republic of China and take an educational course to ensure their strict adherence to and familiarity with the study before they become eligible to treat patients in this study. Those participants allocated to a treatment group will be requested to attend (with their partner or support person if available) a training course and demonstration session by their tutors. The training course involves advice, demonstration of moxibustion, and instructions on how to do moxibustion safely at home. The woman or her partner or support person will be trained to light the moxibustion sticks and to hold an individual stick 2.5 to 3 cm above the two acupoints (SP6 and CV4). The participants will be told not to make the acupoints uncomfortable during treatment. They will be requested to perform this treatment for five to 10 minutes on each acupoint once a day for five to seven consecutive days, and will be supplied with sufficient moxibustion sticks to do so in each treatment period.

#### Rationale for selection of acupoints

The moxibustion treatment protocol to be used is consistent with the principles of traditional moxibustion theories. These principles state that the most common causes of dysmenorrhea in TCM are blood stagnation and cold-damp coagulation in the uterus. According to previous surveys and expert consultations, *Sanyinjiao* (SP6) and *Guanyuan* (CV4) are the most common acupoints used in PD treatment. SP6 is the convergent point of the spleen, liver, and kidney meridians according to TCM, and is closely related to the lower abdomen and uterus [27]. This makes SP6 one of the most commonly used points for gynecologic indications in clinical practice, especially for alleviating dysmenorrhea. The CV4 point in the conception vessel meridian is known as the root of vital *Qi*, and can exert a regulatory effect on the uterus because of its role of providing power to vital movement. Moxibustion at CV4 can treat dysmenorrhea because of its function of warming the conception vessel meridian, tonifying vital *Qi*, and promoting blood circulation to remove blood stasis.

#### Treatment group A (treated before menstruation)

Group A will be treated five days before expected menstrual onset and treatment will continue until menstruation begins. However, the subjects’ menstrual cycles may change and so the actual period of treatment will be five to seven days. The treatment will be the application of a moxa stick (herbal preparation with *Artemisia vulgaris*, Z32021062, Oriental Moxa Co., Suzhou, China) at acupoints SP6 and CV4 to patients in a supine position. The moxa roll is prepared by wrapping mugwort wool (herbal medicines may be mixed in it) with a piece of a paper. It is cylindrical, 1.5 cm in diameter, and 20 cm in length. A lighted moxa roll will be held 2.5 to 3 cm above the acupoint and moved upwards and downwards, to the left and right, or around. The patient should feel warmth but not scorching heat. Each point will be treated with moxibustion for five to 10 minutes.

#### Treatment group B (treated within menstrual period)

Group B treatment will start as soon as menstruation onsets, continuing for five consecutive days. The selection of acupoints and the procedure will be exactly the same as described above for treatment group A.

#### Control group C (waiting list group)

Participants in this group will receive no moxibustion-related intervention while the trial is in process. The subjects in this group will be asked to maintain their normal lifestyle, including diet, exercise, and workload. At the end of the trial, if participants in group C would like to be treated with moxibustion, we will offer free moxibustion treatment five times monthly for three menstrual cycles.

### Outcome assessments

#### Primary outcome measurement

##### Cox Menstrual Symptom Scale (CMSS)

The CMSS has been widely used for integrally evaluating patients’ symptoms [28]. The scale consists of 17 items or symptoms. In the severity evaluation, each symptom is scored via five levels: a score of 0 denotes that the symptom is not noticeable; one denotes it as slightly bothersome; two denotes it as moderately bothersome; three denotes it as severely bothersome; and four denotes it as very severely bothersome. In the duration evaluation, each symptom is scored in five grades: a score of 0 denotes that the symptom did not occur; one denotes that it lasted less than three hours; two denotes that it lasted between three and seven hours; three denotes that it lasted an entire day; and four denotes that it lasted several days. The CMSS will be used in each of the three menstrual cycles of treatment and the three menstrual cycles during the follow-up period.

#### Secondary outcome measurements

##### Visual Analogue Scale

The severity of menstrual pain will be measured with the VAS, a 100-mm horizontal line with endpoints from no pain on the far left to the worst possible pain on the far right. The VAS is scored by measuring in millimeters the distance from the side marked no pain to the edge of the mark made by the patient. Possible scores range from a minimum of 0 to a maximum of 100 mm, as participants will be asked to indicate a perception of pain intensity scored from one to 100. The VAS will be used during baseline measurements and during the first, second, third, fourth, fifth, and sixth menstrual cycle after inclusion.

##### Cox Retrospective Symptom Scale

The Cox Retrospective Symptom Scale (RSS) is a measurement of menstrual symptoms that has been shown to have high reliability, validity, and sensitivity [29]. It gives two scores: a Total Frequency Ratings score (RSS COX1) and an Average Severity Ratings score (RSS COX2). Lower scores indicate better health. The RSS will be used before treatment to assess the baseline condition of the patient.

##### Dysmenorrhea diary

The women will be provided with dysmenorrhea diaries to record the specific time of menstrual pain attacks, the duration of dysmenorrhea, the associated phenomena, and any medication used. This information will be recorded daily during every menstrual cycle of the three study periods. All patients will be carefully instructed on how to use this diary to rate their symptoms. The dysmenorrhea diary will be used during baseline measurements and during the first, second, third, fourth, fifth, and sixth menstrual cycle after inclusion.

##### Self-rating Depression Scale

The Self-rating Depression Scale (SDS) is a 20-item self-reporting questionnaire on the symptoms of depression. Subjects rate each item according to how they felt during the preceding seven days. Item responses are ranked from one to four. The sum of the 20 items produces a score between 20 and 80, with a score of greater than 40 suggesting clinically relevant depression [30]. The SDS will be used on the day when menstruation ends in the last month of the baseline period, and in the fourth menstrual cycle after inclusion (after completion of three sessions of treatment).

##### Self-rating Anxiety Scale

The Self-rating Anxiety Scale (SAS) has been widely used in research and in clinical practice for the detection of anxiety [31]. It also consists of 20 items rated on numerical values of one to four. The total SAS score may vary from 20 (no anxiety at all) to 80 (severe anxiety), with a value of greater than 40 suggesting the presence of a clinically relevant anxiety disorder. The SAS will be used on the day when menstruation ends in the last month of the baseline period, and in the fourth menstrual cycle after inclusion (after completion of three treatment sessions).

### Follow-up

The follow-up assessment is designed to evaluate the long-term effects of moxibustion. Upon completion of treatment, follow-up assessments will be conducted at the fourth, fifth, and sixth menstrual cycle after inclusion, lasting three menstrual cycles. In addition, patients will keep the daily dysmenorrhea diary and complete the CMSS and VAS measurements during every menstrual cycle in the follow-up period. Patients will send the relevant information to researchers immediately via email or short message service. To encourage participant compliance, we will show special solicitude for every participant and closely monitor the evolution of their illness.

### Statistical analysis plan

#### Data integrity

No record can be missed or omitted in the original data source. Any corrections should be explained in the appended notes signed and dated by the physicians participating in the clinical trial; primary entries are not allowed to be changed. After the observation of a clinical case, case report form (CRF) files will be submitted to the project directors for verification and signing by supervisors.

Data input will be done separately by two data collectors with computers and locked once the checking work is done. The data manager will clarify any questions regarding the CRF with the researchers through clinical supervisors. Researchers should answer any questions as soon as possible so that the data manager can conduct the modification, validation, and entry of the data.

#### Analysis

A statistician blinded to the whole trial process will perform the statistical analysis using the SAS statistical software package (Version 9.1, SAS Institute Inc.) in the Computer Integrated Manufacturing System. For the evaluation of curative effect in this trial, the per protocol set (PPS) will be used. The full analysis set (FAS) is determined according to an intention-to-treat population; all patients who are randomized and receive at least one treatment session will be included in the analysis set. The PPS is defined as the patients who complete the study and do not have major protocol violations. Demographic data and other basic indicators will be analyzed to test the balance of the three groups at baseline. The main indicators and global indicators will be analyzed within the FAS and PPS. The results will be described with the mean, standard deviation, median, P25 (Percentile 25), P75 (Percentile75), maximum, and minimum values of the differences between the treatment period and the baseline period. Between-group differences will be tested using repeated measure analyses of variance. The accepted level of significance for all analyses was *P* <0.05. Considering that the efficacy might be affected by factors such as age, age of menarche, and course of disease, all factors will be considered as co-variants for covariance analysis or logistic regression analysis when comparisons among groups are made. Missing data will be replaced according to the principle of multiple imputations.

### Safety monitoring

Possible adverse events (AEs) due to moxibustion include blisters, redness, itching, burns, and respiratory symptoms. All unexpected and unintended responses will be reported as AEs by the researcher at every visit, even if they are not necessarily related to moxibustion intervention. The AEs will be carefully recorded in the CRF by the corresponding research staff.

## Discussion

This proposed trial is a randomized, controlled clinical trial with separation of the practitioner, assessor, and statistician. The aims of this trial are to evaluate the efficacy and safety of moxibustion for treating PD, and to identify the optimal interventional time of moxibustion for PD treatment.

The form of moxibustion used in this trial will be suspended moxibustion treatment. When suspended moxibustion is applied to acupoints, the patient will have non-local or non-superficial heat sensation, such as penetrating heat, expanding heat, and transmitting heat, as *DeQi* appears. Selection of optimal acupoints for PD plays an important role in moxibustion treatment effectiveness. In practice, the treatment effect of moxibustion is affected by many other influential factors, including the intensity of heat stimulation, the ingredients of the moxa pillar, and the moxibustion sensations experienced. The most important factor contributing to the effectiveness of moxibustion is thought to be the selection of proper interventional time. However, in existing studies, interventional timing of moxibustion was not included as a key variant to be assessed.

In another undergoing study, we mainly focused on the effectiveness of moxibustion for pain relief in PD, which can be referred to the published protocol [32]. As to the current one, it is conceived on the basis of the former one. As indicated by the results from the initial analysis, moxibustion is effective for PD pain management; thus we want to further assess the impact of intervention time of moxibustion on the outcome of treating PD, so as the physiological tests’ change by treatment. In clinical practice, moxibustion treatment for PD can also be differed by the intervention timing in China, and there is no relative study on this focus. Thus we carried out the present study as a continuum which is reported by the current protocol. It is anticipated that the result may demonstrate the optimal interventional timing for PD and further extend our understanding of intervention from a chrono-therapeutic perspective, which will be highlights of our further reports. Moreover, the present study that was designed in accordance with the Consolidated Standards of Reporting Trials (CONSORT) statement, and guidelines will provide useful information to guide the clinical practice of moxibustion for PD.

In addition, our study may also have the following strengths which will be potential points of discussion in the final reports. The dysmenorrhea diaries will be used to record the symptoms and management of menstrual pain in every menstrual cycle; these diaries are important for quality monitoring of the recurrence of new PD episodes. Besides the evident physical concerns, PD may be particularly disruptive to adolescent girls and women who experience mood fluctuations, such as depression or anxiety. There is increasing evidence that psychological disorders coexist with dysmenorrhea, and these conditions may synchronize with menstrual pain. The question remains whether anxiety and depression make women prone to suffering from dysmenorrhea, or whether dysmenorrhea causes anxiety and depression. We believe that women with mood changes tend to have lowered pain thresholds compared with mentally healthy women. Anxiety and depression disrupt patients’ experiences, expressions, behaviors, and pain-coping responses. Therefore, a better understanding of the relationship between psychological characteristics and PD is crucial to the assessment of the effectiveness of moxibustion as a treatment for PD. Hence, we will apply the SAS and the SDS to examine the link between PD and psychological symptoms of depression and anxiety among dysmenorrhea sufferers.

On the other hand, there are also limitations to this study. Concealment is the most critical factor in the allocation process, with a high risk of bias possibly generated by unconcealed allocation [33]. One limitation of this study is the possibility of a high risk of bias regarding blinding, as the waiting list group will be used as a control instead of a group receiving a sham procedure. Another limitation is that as moxibustion treatment will begin five to seven days before menstruation or at the onset of pain, the treatment may interfere with the menstrual period; treatment may cause menstruation to occur one or two days sooner or later than the usual cycle. Therefore, it may be difficult to predict an exact menstruation start date for the patients, and this may affect the therapeutic effect.

It is believed that this trial will provide good evidence to demonstrate the efficacy and safety of moxibustion for patients with PD. Moreover, it will be of great significance for clinical practice to identify an optimal moxibustion treatment time for PD.

## Trial status

The trial is currently in the recruitment phase. Participant recruitment started in June 2014, and is expected to end in September 2015.

## Abbreviations

CMSS: Cox Menstrual Symptom Scale
CRF: Case report form
FAS: Full analysis set
IRB: Institutional Review Board
PD: Primary dysmenorrhea
PPS: Per protocol set
RCT: Randomized controlled trial
RSS: Cox Retrospective Symptom Scale
SAS: Self-rating Anxiety Scale
SDS: Self-rating Depression Scale
TCM: Traditional Chinese Medicine
VAS: Visual analogue scale
